# Fusion of Mitochondria to 3-D Networks, Autophagy and Increased Organelle Contacts are Important Subcellular Hallmarks during Cold Stress in Plants

**DOI:** 10.3390/ijms21228753

**Published:** 2020-11-19

**Authors:** Philip Steiner, Othmar Buchner, Ancuela Andosch, Gerhard Wanner, Gilbert Neuner, Ursula Lütz-Meindl

**Affiliations:** 1 Department of Biosciences, Faculty of Natural Sciences, University of Salzburg, Hellbrunnerstraße 34, A-5020 Salzburg, Austria; mail@o.buchner.co.uk (O.B.); ancuela.andosch@sbg.ac.at (A.A.); ursula.luetz-meindl@sbg.ac.at (U.L.-M.); 2 Ultrastructural Research, Department Biology I, Faculty of Biology, Ludwig-Maximilians-University, Großhadernerstraße 2−4, Planegg-Martinsried, D-82152 Munich, Germany; wanner@lrz.uni-muenchen.de; 3 Department of Botany, Functional Plant Biology, Faculty of Biology, University of Innsbruck, Sternwartestraße 15, A-6020 Innsbruck, Austria; gilbert.neuner@uibk.ac.at

**Keywords:** electron microscopy, TEM, FIB-SEM, *Micrasterias denticulata*, *Lemna* sp., *Ranunculus glacialis*, organelle networks, ultrastructure, freezing stress

## Abstract

Low temperature stress has a severe impact on the distribution, physiology, and survival of plants in their natural habitats. While numerous studies have focused on the physiological and molecular adjustments to low temperatures, this study provides evidence that cold induced physiological responses coincide with distinct ultrastructural alterations. Three plants from different evolutionary levels and habitats were investigated: The freshwater alga *Micrasterias denticulata*, the aquatic plant *Lemna sp.*, and the nival plant *Ranunculus glacialis.* Ultrastructural alterations during low temperature stress were determined by the employment of 2-D transmission electron microscopy and 3-D reconstructions from focused ion beam–scanning electron microscopic series. With decreasing temperatures, increasing numbers of organelle contacts and particularly the fusion of mitochondria to 3-dimensional networks were observed. We assume that the increase or at least maintenance of respiration during low temperature stress is likely to be based on these mitochondrial interconnections. Moreover, it is shown that autophagy and degeneration processes accompany freezing stress in *Lemna* and *R. glacialis*. This might be an essential mechanism to recycle damaged cytoplasmic constituents to maintain the cellular metabolism during freezing stress.

## 1. Introduction

Low temperatures, such as chilling and freezing stress [1,2,3,4], have a severe impact on the distribution, physiology, and survival of plants (see references [5,6,7] and others). In contrast to chilling, where low temperature solely affects the plants, during freezing water turns into ice. While intracellular ice formation is lethal for plant cells [8], many plants are able to transiently endure extracellular ice [6,9,10,11]. When plants are exposed to sub-zero temperatures in nature, they can hardly escape and thus have to develop frost survival mechanisms such as freezing avoidance [10,12] and freezing tolerance [11,13].

Previous studies on diverse plant systems provided information on physiological responses [6,9,14] as well as molecular mechanisms during cold stress. Molecular adjustments include alterations in lipid and sugar composition but also in expression of compounds (proteins/genes) and transcription factors [12,15,16,17,18]. Especially during freezing stress, several plants produce or activate antifreeze proteins in order to support freezing tolerance and avoid intracellular freezing [11,13,19,20].

Despite our knowledge of these molecular and physiological responses, it is unknown whether the rapid activation and changes in metabolism, occurring as a consequence of cold stress, are co-occurring with structural reorganizations within the cells. Since structure represents the basis for plant physiology, ultrastructural investigations on cytoplasmic organization and morphology of organelles in combination with physiological assays, as those provided in the present study, may provide important information for the fundamental understanding of plant responses during low temperature stress.

It is known from several observations (see references [21,22,23] and others), that cell organelles and compartments may alter and interact structurally during stress. In a recent study [24], structural changes of the cell wall and alterations in organelle distribution were observed in the early branching streptophyte alga *Klebsormidium crenulatum* (Klebsormidiophyceae) during freezing stress. In the late branching streptophyte alga *Micrasterias* (Zygnematophyceae, Desmids) for example, the fusion of mitochondria to local networks was observed during ionic stress [22] and the degeneration of dictyosomes occurred as a consequence of cadmium stress [25]. However, only few studies have focused on structural alterations of organelles in plant cells during cold stress so far [5,7,26].

The occurrence of autophagy and degradation processes have been reported from different plant cells during stress and seem to be required for limiting stress induced cytoplasmic damage [27]. In *Micrasterias,* autophagy is induced for example during salt stress [28] or under the impact of heavy metals such as cadmium [29].

The visualization of mitochondrial dynamics and interactions with other organelles, by means of life-imaging methods with specific tracking dyes such as Mito-Trackers, has been successfully performed in several studies before [21,30,31]. These methods are highly adequate to investigate organelle dynamics. For providing sufficient resolution, e.g., for discriminating between real membrane fusions or simple surface contacts of adjacent organelles [32] and for the depiction of structural changes within organelles or degradation processes, it is indispensable to investigate subcellular structural reactions during low temperature stress by means of high-resolution, nano-scale electron microscopic methods after cryo-preservation. The freezing of plant tissue prior to high pressure freezing (HPF) was enabled by the development of an automatic freezing unit (AFU) for subsequent electron microscopic investigations [33].

In this study, we investigate plant model systems of diverse evolutionary levels from algae to higher freshwater and land plants with respect to their cellular responses to low temperature stress. Our main model system, the unicellular freshwater alga *Micrasterias denticulata* is closely related to higher land plants [34,35]. The alga measures up to 200 µm in diameter and has correspondingly large organelles. It inhabits peat bogs, at elevations up to above 3000 m [36], which makes it highly adequate for investigations on cold stress responses. Due to the numerous data that are already available on cellular and subcellular stress responses in *Micrasterias* [29,37,38,39,40,41], the main focus of this study is placed on this model system. Additionally, comparative experiments were performed with the higher plants *Lemna* sp. and *R. glacialis* in order to prove the generality of the results obtained in *Micrasterias*. The higher freshwater plant *Lemna* has already been used as a model system in different kinds of investigations [42,43] and also for comparison with *Micrasterias* [22]. *R. glacialis* is a nival plant, inhabiting sites above 2000 m elevation in the European Alps (up to 4275 m), and has been subjected to several cold stress related physiological and ultrastructural investigations [44,45,46,47,48,49,50]. The selected set of plant model systems originating from diverse habitats and belonging to various evolutionary levels is intended to provide a comprehensive insight into the structural responses of plant cells to cold stress.

Since 2-dimensional electron microscopy is insufficient for depicting 3-dimensional structural alterations or organelle contacts and fusions [22,24,51], we combine 2-D transmission electron microscopic (TEM) methods with 3-D focused ion beam-scanning electron microscopy (FIB-SEM) in the present study. The structural data on cellular responses and rearrangements during low temperature stress obtained by these methods are correlated to functional parameters determined by in-vitro assays (respiration, photosynthesis). This combination of methods has already proven to be of high interpretive value in previous studies [22,25,41,52].

## 2. Results

### 2.1. Fusion and Aggregation of Mitochondria to Local Networks during Cold Stress in Micrasterias

**Figure 1:**
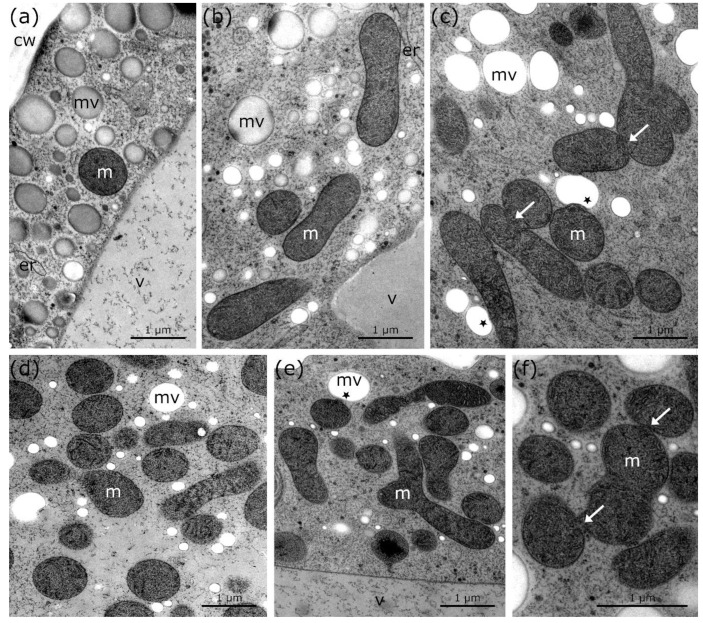
TEM micrographs of mitochondria in *Micrasterias denticulata* cells during cold stress in comparison to controls at +20 °C. (**a**) Control at +20 °C with round, solitary mitochondrion and single mucilage vesicles. (**b**) +4 °C, 24 h chilling treatment with elongated mitochondria and slightly bloated endoplasmic reticulum. (**c**) +4 °C, 3 weeks treatment with elongation, aggregation, and fusion of mitochondria (arrows) and protrusion of mitochondria into mucilage vesicles (asterisks). (**d**) −2 °C freezing (without ice) treatment—elongation and aggregation of mitochondria visible. (**e**) −2 °C, extracellularly frozen cell with elongation and aggregation of mitochondria and protrusion of mitochondria into mucilage vesicle (asterisk). (**f**) Higher magnification of −2 °C, extracellularly frozen cell shows attachment and fusion of outer mitochondrial membrane (arrows) as well as aggregation to mitochondrial network. m: mitochondria, mv: mucilage vesicles, v: vacuole, cw: cell wall, er: endoplasmic reticulum.

**Figure 2:**
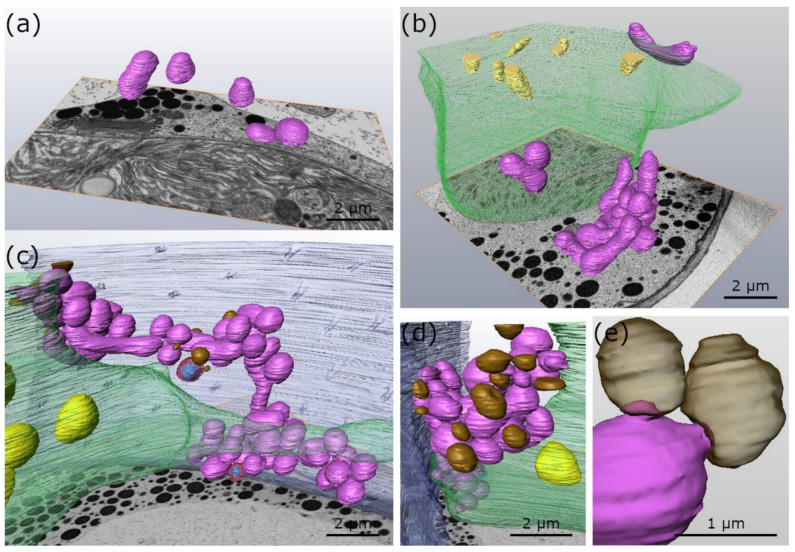
3-D FIB-SEM reconstructions of organelles of *Micrasterias denticulata* during low temperature stress in comparison to controls at +20 °C. (**a**) Control at +20 °C with single unfused mitochondria (**b**) −2 °C freezing (without ice) treatment. Mitochondria aggregated and fused to single mitochondrial clusters. (**c**) −2 °C, extracellularly frozen cell, with large mitochondrial network. (**d**) −2 °C, extracellularly frozen cell, with mitochondrial network in contact with mucilage vesicles. (**e**) Higher magnification of −2 °C, extracellularly frozen cell depicts protrusion of mitochondria into transparent mucilage vesicles. purple: mitochondria, green: chloroplast, (transparent) brown: mucilage vesicles, transparent red with blue crystal: peroxisomes, yellow: starch grains, blue: cell wall with cell pores.

2-D TEM imaging in combination with 3-D FIB-SEM reconstructions show that the mitochondria of untreated *Micrasterias* cells, grown at +20 °C, are spherically shaped and solitary distributed in the cytoplasm (Figure 1a and Figure 2a; Table 1). In *Micrasterias*, mitochondria begin to elongate and appear in close proximity to each other during short-term (24 h) chilling stress at +4 °C (Figure 1b; Table 1). During long-term (3 weeks) chilling stress at +4 °C, mitochondria start to aggregate and fuse with one another (Figure 1c arrows; Table 1). With decreasing temperatures from −2 °C freezing (without ice) to −2 °C extracellular freezing stress, mitochondria of *Micrasterias* aggregate to local networks and their outer membranes are attached or fuse with each other (Figure 1d–f and Figure 2b–d; arrows; Table 1). The single networks are dispersed in the cytoplasm and are not in contact with other networks (Figure 2c; Table 1). In Figure 2c, all depicted mitochondria are fused and aggregated to one large mitochondrial network. Our 3-D FIB-SEM and 2-D TEM investigations provide evidence that the mitochondrial contacts, observed during −2 °C freezing without ice (Figure 1d and Figure 2b; Table 1) and −2 °C freezing stress (Figure 1e,f and Figure 2c,d; Table 1), represent in fact fusions of mitochondria to local networks. Such fusions and aggregations are not present in controls of *Micrasterias,* grown at +20 °C (Figure 1a and Figure 2a; Table 1). Furthermore, protrusions of the outer mitochondrial membrane into mucilage vesicles appear during chilling and freezing stress in *Micrasterias* (Figure 1c,e and Figure 2c–e; asterisks; Table 1), indicating functional interactions.

**Figure 3:**
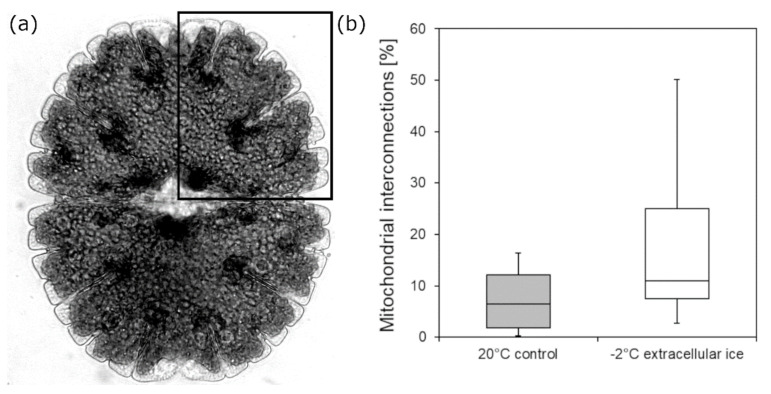
(a) Schematic depiction of the analyzed area in Micrasterias. (b) Mitochondrial fusions and contacts (%) in Micrasterias denticulata during extracellular freezing stress at −2 °C in comparison to controls at +20 °C determined by analysis of TEM micrographs (n = 10). Mean values were not significantly different (p = 0.069; t-test). Boxes indicate the median (horizontal line inside the box) and the 25th and the 75th percentile (bottom and top border). Whiskers indicate maxima and minima and extend maximum to 1.5 times box-height.

TEM analysis of *Micrasterias* cell quarters (Figure 3a; see section Material and Methods) shows that in −2 °C extracellularly frozen *Micrasterias* cells, the number of mitochondrial contacts and fusions to aggregating networks is apparently higher than in controls at +20 °C (Figure 3b). Nevertheless, the observed difference is not statistically significant (*p* = 0.069).

### 2.2. Mitochondrial Networks and Autophagy in Lemna during Cold Stress

**Figure 4:**
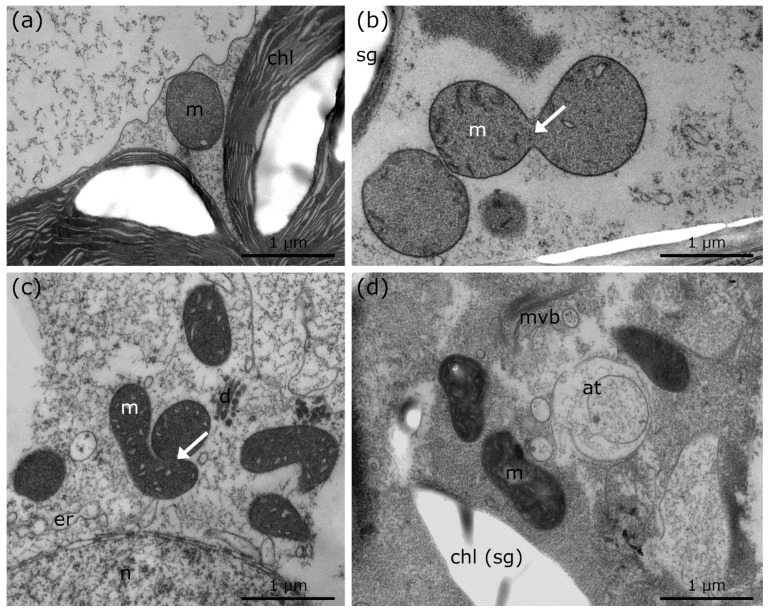
TEM micrographs of *Lemna* sp. during cold stress in comparison to controls at +20 °C. (**a**) +20 °C control of *Lemna* sp., with single, round mitochondrion. (**b**) +4 °C, 24 h chilled *Lemna* sp. depicting mitochondrial fusion (arrow). (**c**) +4 °C, 3 weeks chilled *Lemna* sp. with mitochondrial aggregation, fusion (arrow) and bloated ER. (**d**) −2 °C, extracellularly frozen *Lemna* sp. with signs of degradation (chloroplast envelope dissolved; autophagic structures), mitochondrial elongation and increased number of multi vesicular bodies. at: autophagic structures, m: mitochondria, mvb: multi vesicular body, er: endoplasmic reticulum, chl: chloroplast, sg: starch grain, d: dictyosome, n: nucleus.

Controls of *Lemna* at +20 °C show round mitochondria, which are randomly distributed in the cytoplasm (Figure 4a; Table 1). During chilling and freezing stress, elongations, aggregation, and membrane fusions of mitochondria are visible in *Lemna* (Figure 4b–d, arrows; Table 1). Although mitochondrial aggregation and fusions to networks in *Lemna* are not as pronounced as those in *Micrasterias* during cold stress, the appearance of the mitochondrial fusion to networks is similar to that in *Micrasterias*, indicating that mitochondrial aggregation occurs in the same way. Furthermore, mitochondrial alterations are accompanied by autophagic structures during freezing stress in *Lemna* (Figure 4d; Table 1).

### 2.3. Ultrastructural Alterations in Palisade Parenchyma Cells of R. glacialis During and after −5 °C Extracellular Freezing with Additional Recovery to +10 °C and Comparison to Controls at +10 °C

**Figure 5:**
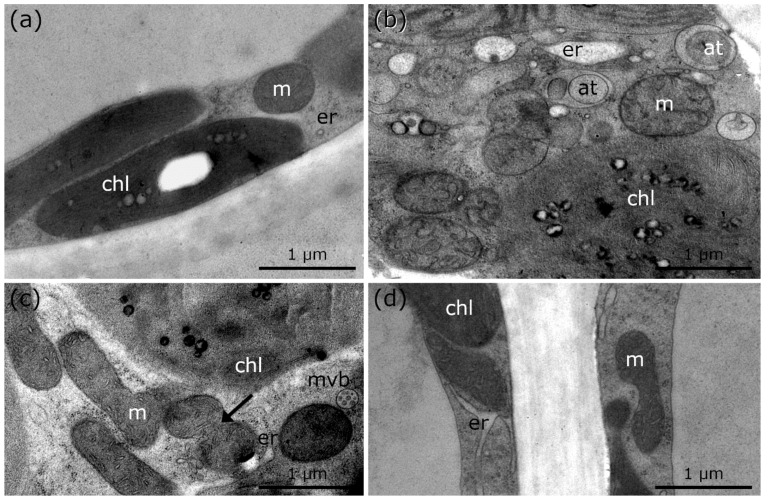
TEM micrographs of palisade parenchyma cells of *R. glacialis* during and after cold stress in comparison to controls at +10 °C. (**a**) Control of *R. glacialis* at +10 °C with single mitochondrion. (**b**) −5 °C extracellularly frozen *R. glacialis* with aggregated mitochondria, bloated ER and numerous autophagic structures. (**c**) *R. glacialis* at +10 °C, 15 min after −5 °C extracellular freezing stress. Mitochondrial fusion and aggregation (arrow), minor bloating of ER, and multi vesicular bodies are clearly visible. (**d**) Recovery of *R. glacialis* at +10 °C, 24 h after −5 °C extracellular freezing stress. Minor structural alterations of mitochondria and ER still visible. No autophagic structures were observed. at: autophagic structures, m: mitochondria, mvb: multi vesicular body, er: endoplasmic reticulum, chl: chloroplast.

Controls of *R. glacialis* at +10 °C show single, round mitochondria and regularly shaped ER cisternae (Figure 5a; Table 1), similar to the controls of *Micrasterias* and *Lemna* at +20 °C (Figure 1a and Figure 4a; Table 1). During freezing stress at −5 °C, mitochondria aggregate and fuse to networks but appear structurally disintegrated. Frequent autophagic structures and bloated ER are observed (Figure 5b; Table 1). After rapid thawing and rewarming to +10 °C within 15 min, minor recovery of cell structure and organelles occurs but mitochondria remain fused and aggregated to networks (Figure 5c, arrows; Table 1). After subsequent recovery at +10 °C for 24 h, a progressive regeneration of cell structure and organelles is observed (Figure 5d; Table 1). Experiments carried out with leaves of *R. glacialis* (Figure 5a–d) demonstrate that compartments and organelles regain their regular cellular structure within 24 h of recovery period. Mitochondrial and ER alterations are slightly maintained but autophagic compartments are not visible (Figure 5d; Table 1).

### 2.4. Other Structural Alterations during Freezing Stress in Micrasterias, Lemna and R. glacialis in Comparison to Controls

**Figure 6:**
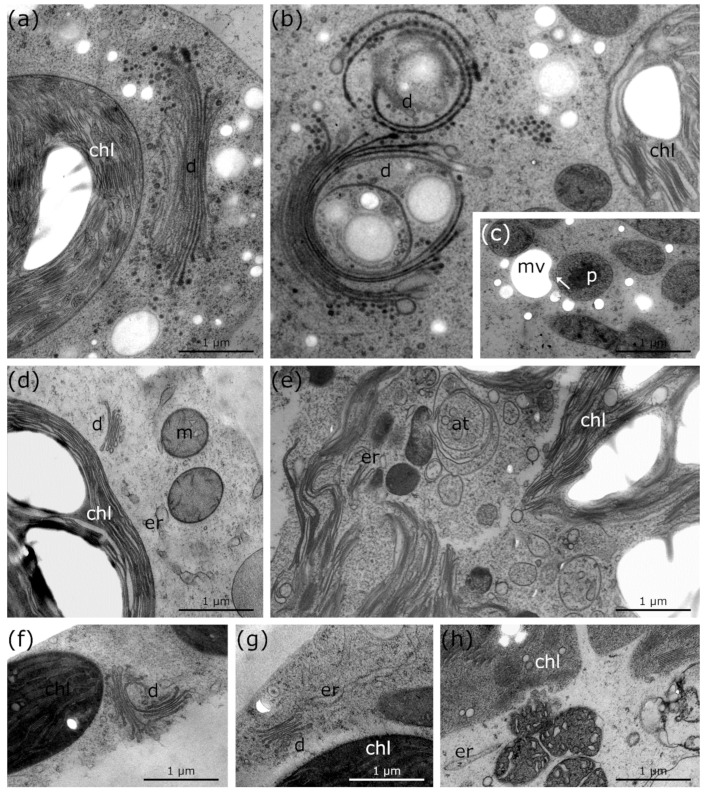
TEM micrographs of *Micrasterias denticulata*, *Lemna* sp. and palisade parenchyma cells of *Ranunculus glacialis* during freezing stress in comparison to controls at +20 °C. (**a**) *Micrasterias* control at +20 °C with regular thylakoid structure of chloroplast and distinct dictyosome shape and number of cisternae. (**b**) *Micrasterias* during freezing stress at −2 °C with degrading dictyosomes and bloated thylakoids. (**c**) *Micrasterias* during freezing stress at −2 °C with protrusion of peroxisome into mucilage vesicle (arrow). (**d**) *Lemna* control at +20 °C with regular thylakoid structure of chloroplast, distinct size and shape of dictyosome and solitary distributed, round mitochondria. (**e**) *Lemna* during −2 °C freezing stress with bloated thylakoids, enlarged ER and autophagic structures. (**f**) *R. glacialis* control at +10 °C with dictyosome in division and regular thylakoid structure. (**g**) *R. glacialis* control at +10 °C with regular dictyosome, regular thylakoid and ER structure. (**h**) *R. glacialis* during freezing stress at −5 °C with degraded thylakoids and enlarged ER structure. at: autophagic structures, chl: chloroplast, d: dictyosome, er: endoplasmic reticulum, *p*: peroxisome, m: mitochondria, mv: mucilage vesicles.

Untreated controls of *Micrasterias* at +20 °C have a defined number of 11 dictyosomal cisternae (Figure 6a; Table 1); see also [39]. The size, shape, and number of cisternae as well as vesicle production does not seem to be affected until freezing is induced. Freezing stress causes degradation of cisternae and a reduction of vesicle production (Figure 6b; Table 1). In comparison to *Micrasterias* controls (Figure 1a and Figure 6a; Table 1), ER cisternae and thylakoids of chloroplasts become bloated/enlarged with decreasing temperature (Figure 1b and Figure 6b; Table 1). Furthermore, protrusions of peroxisomes into mucilage vesicles appear during freezing stress in *Micrasterias* (Figure 6c; arrow; Table 1). These protrusions seem to resemble the protrusions of mitochondria into mucilage vesicles, which are also observed during freezing stress in *Micrasterias* (Figure 1c,e and Figure 2e; Table 1).

Controls of *Lemna* at +20 °C show smaller dictyosomes (Figure 6d; Table 1) than Micrasterias (Figure 6a; Table 1) but with the same distinct shape. When freezing stress was induced in *Lemna,* dictyosomal cisternae became strongly degraded and the distinct shape was no longer visible (Figure 4d and Figure 6e; Table 1). The chloroplast envelope dissolves after ice nucleation during exposure to freezing stress and enlarged thylakoid membranes and starch grains remain solitary dispersed inside the cytoplasm (Figure 4d and 6e; Table 1) in comparison to an intact chloroplast in controls at +20 °C (Figure 4a and Figure 6d; Table 1).

During freezing stress in *R. glacialis*, ER cisternae are slightly enlarged (Figure 6h; Table 1), compared to controls at +10 °C (Figure 6g; Table 1). Furthermore, the thylakoid structure and the dictyosomes degraded during −5 °C freezing stress (Figure 6h; Table 1) in comparison to controls (Figure 6f; Table 1). Most of these processes are reversible in *R. glacialis* after thawing and rewarming the plants to +10 °C within 24 h (Figure 5d; Table 1).

### 2.5. Apparent Photosynthesis and Dark Respiration of Micrasterias and Lemna at +4 °C in Comparison to Controls at +20 °C

**Figure 7:**
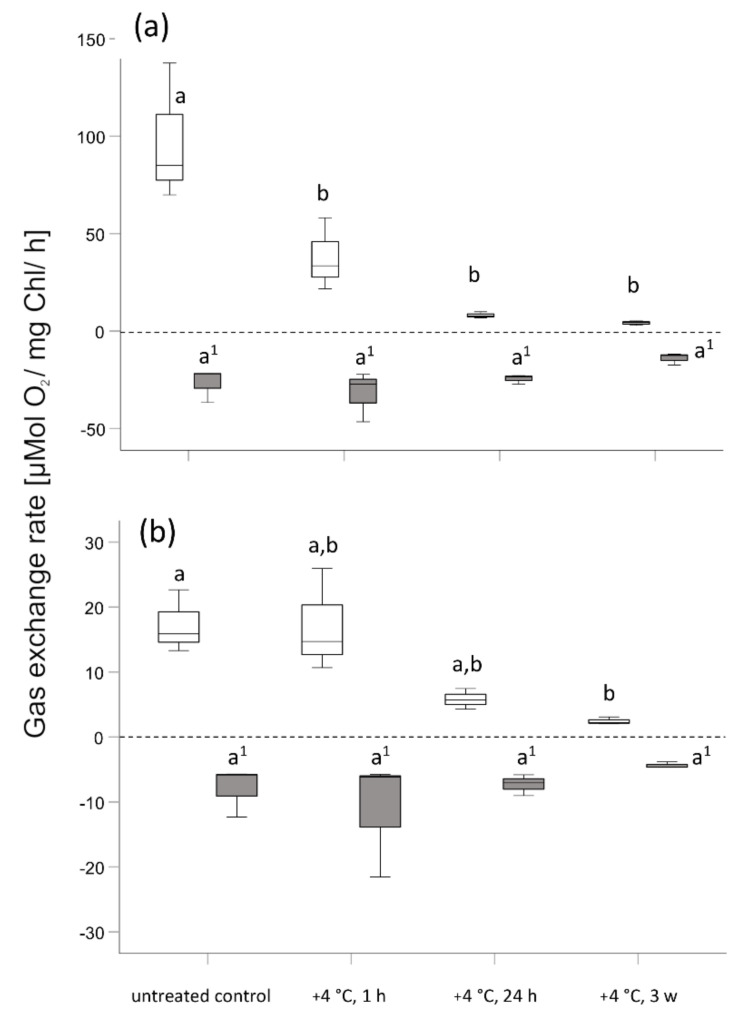
Photosynthetic oxygen production (apparent photosynthesis, open boxplots) and dark respiration (grey boxplots) of (**a**) *Micrasterias denticulata* and (**b**) *Lemna* sp. after 1 h, 24 h, and 3 weeks of chilling stress at +4 °C. Each boxplot relates to 3 independent biological replicates (*n* = 3). Different letters (a, b) indicate significant differences between means (*p* < 0.05). Letters with superscript numbers (a’, b’) are related to the dark respiration rate R_d_. (One-way ANOVA followed by Duncan’s and Games Howell’s test). Boxes indicate the median (horizontal line inside the box) and the 25th and the 75th percentile (bottom and top border). Whiskers indicate maxima and minima and extend maximum to 1.5 times box-height.

Polarographic oxygen determination in *Micrasterias* (Figure 7a) indicates the maintenance of, and even depicts a slight increase of, dark respiration (R_d_) mean values during low temperature chilling stress (+4 °C) after 1 h in comparison to controls at +20 °C. After 24 h, when mitochondria start forming networks (Figure 1b; Table 1), R_d_ is still maintained. After 3 weeks exposure to +4 °C, mean R_d_ rate decreases to approximately half of that of control values at +20 °C. However, respiration and mitochondrial networks are still maintained. Polarographic oxygen determination in *Lemna* (Figure 7b) displays a similar change in R_d_ as in *Micrasterias* in response to exposure to +4 °C, when mitochondria fuse and aggregate to networks and respiration is still maintained (Figure 4b–d; Table 1). No significant change of R_d_ (*p* > 0.05) is measured for 3 weeks of exposure to a chilling stress at +4 °C in *Micrasterias* and *Lemna*. Apparent photosynthesis in *Micrasterias* (Figure 7a) significantly decreases (*p* < 0.05) after 1 h of +4 °C chilling stress and extended exposure to +4 °C. In comparison to controls at +20 °C, apparent photosynthesis in *Lemna* (Figure 7b) initially remains (after 1 h at +4 °C) but decreases significantly (*p* < 0.05) with prolonged exposure to +4 °C.

### 2.6. Dark respiration of R. glacialis Leaves before and after Freezing Stress at −5 °C

**Figure 8:**
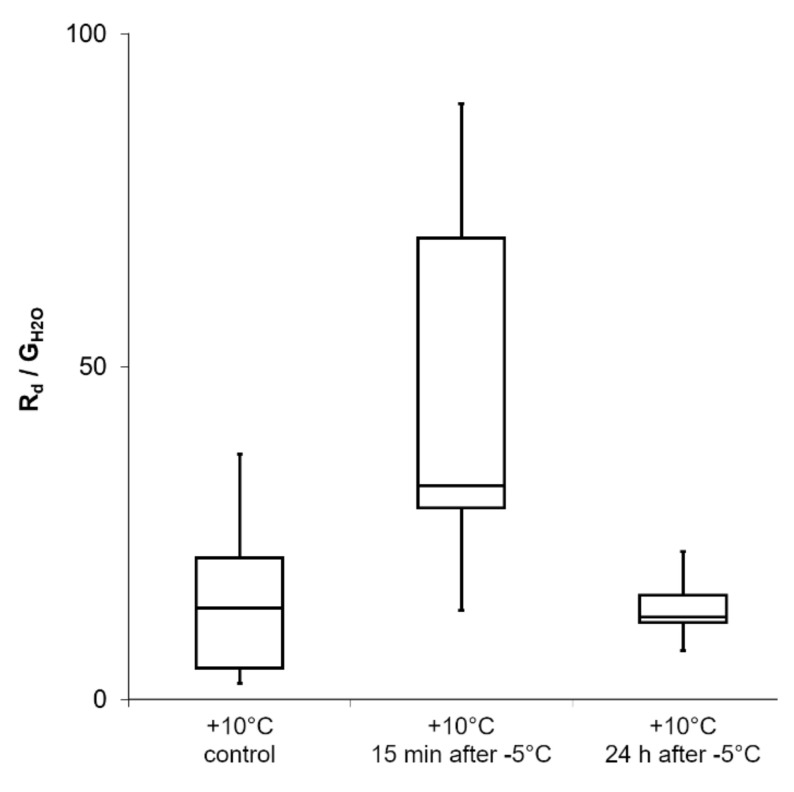
Gas exchange measurements during a freezing and thawing (recovery) experiment on *R. glacialis* leaves. The Boxplots show the dark respiration rate (R_d_) in dependence on the diffusive conductance rate (G_H20_). The mean value is increased but without statistical significance (*p* > 0.05; repeated measures ANOVA with Bonferroni-correction). Boxes indicate the median (horizontal line inside the box) and the 25th and the 75th percentile (bottom and top border). Whiskers indicate maxima and minima and extend maximum to 1.5 times box-height.

Dark respiration rate in dependence on diffusive conductance (R_d_/G_H2O_) measured on *R. glacialis* leaves (Figure 8) was apparently but not significantly (*p* > 0.05) increased when determined at +10 °C 15 min after exposure to freezing stress at −5 °C. 24 h after freezing at −5 °C, when *R. glacialis* leaves recovered at +10 °C, mean R_d_/G_H2O_ equals control values.

## 3. Discussion

The observed cellular and subcellular responses to sublethal chilling and freezing stress were generally comparable in the three different plants, which provides evidence for the general structural alterations of organelles and cytoplasmic organization. Particularly, an increase in organelle contacts and most prominently, the fusion of mitochondria to extended local networks was identified by means of high-resolution 2-D and 3-D electron microscopy. Mitochondrial fusion to local networks was most prominently pronounced in the large *Micrasterias* cells. Tendencies towards mitochondrial fusion, such as elongation and network formation together with clear signs of autophagy, were also observed in *Lemna* and *R. glacialis.* All structural alterations increased with the severity of temperature stress. However, the respiratory capacity remained almost unaltered and the photosynthetic efficiency was still maintained during chilling stress.

Among all organelles, mitochondria seem to play an important role in management of cold and freezing stress. Morphological alterations of mitochondria, such as aggregation or changes of (inner) membrane structure and permeability, were described as primary markers for general stress in animal and plant cells in previous studies, before cells undergo cell death [21,53,54]. Mitochondria of *Cucurbita pepo*, for example, elongate and fuse to mitochondrial reticuli after anaerobe stress [23]. Similar mitochondrial alterations were observed in the lace plant *Aponogeton madagascariensis,* where the plant produces perforations in its leaves during PCD (programmed cell death ) [21]. In the large cells of *Micrasterias,* it was shown that transient mitochondrial fusion to local networks is essential for ionic and osmotic stress management [22,28]. We now observe a similar stress response during chilling and freezing stress in this alga but also in *Lemna* and *R. glacialis*, which sustain even lower temperatures. This indicates that mitochondrial fusion seems to be a general stress response that represents the structural basis for maintenance and/or increase of respiration during low temperature stress. It is assumed that this occurs by interconnecting the respiratory chains and by enhancing the buffer capacity against ionic imbalances due to stress [22]. This is particularly important in cases where repair mechanisms require increased energy supply. Furthermore, the membrane contacts of mitochondria with mucilage vesicles are also increased during cold and freezing stress in *Micrasterias*. Mitochondria and mitochondrial networks form protrusions into the mucilage vesicles, indicating functional interactions. It is known that *Micrasterias* cells can rapidly excrete degraded cell constituents or toxic substances by transferring them into mucilage vesicles, which are released at the cell surface [55]. These interactions between mitochondria and mucilage vesicles may thus also be favorable during low temperature stress.

In *R. glacialis* and *Lemna*, degrading organelles and the occurrence of autophagosomes were frequently observed during freezing stress. Previous studies reported that particularly autophagy, but also other degradation processes, are essential for limiting the cytoplasmic damage due to stress [27,56]. This seems to be also the case during freezing stress in *R. glacialis* and *Lemna*. In *R. glacialis* leaves, most prevalent structural alterations that were observed during extracellular freezing were reversed after 24 h recovery at +10 °C. Only minor alterations of mitochondria and ER remained, yet after 24 h recovery no autophagic structures could be detected in the cytoplasm anymore. This shows a good recovery capacity of *R. glacialis* after freezing events. Rapid recovery seems important, as night frosts are frequent throughout the whole vegetation period in its nival habitat [57,58,59] and ice nucleation with consequent freezing cytorrhysis of mesophyll cells is already observed at −2.6 °C [50]. Mitochondrial fusion and aggregation in *R. glacialis* appeared during and directly after extracellular freezing stress at −5 °C. This matches well with the increased mean values of respiration that were measured 15 min after thawing from −5 °C. As we investigated the palisade parenchyma cells of *R. glacialis*, we did not detect any structures that could be related to the triglycerides found close to the plasmalemma of spongy parenchyma cells [50].

The bloating of thylakoids as consequence of cold and freezing stress was observed in *Micrasterias* and *Lemna* when photosynthetic efficiency was maintained during cold stress in both organisms. Morphological alterations of the whole chloroplast structure and the outer chloroplast membrane were also reported during different stress scenarios such as virus infections [60] or cold stress [61]. Dictyosomes were not visibly affected in structure and function during chilling and freezing stress (without ice). However, degraded cisternae of dictyosomes were found during freezing stress in *Micrasterias, Lemna* and *R. glacialis*. ER cisternae also respond uniformly in all three plants by enlarging and bloating with decreasing external temperatures. Since this concerned in particular the rough ER cisternae, it is likely that protein synthesis increases during stress, but it may also be involved in maintaining the ionic balance of the cytoplasm during low temperature stress. Furthermore, protrusions of peroxisomes into mucilage vesicles were observed in *Micrasterias* cells during freezing stress. The contact between the two organelles appears similar to the protrusions of mitochondria into mucilage vesicles in *Micrasterias* during freezing stress. We therefore assume similar functional interactions of peroxisomes and mucilage vesicles in *Micrasterias* during freezing stress (see above [55]).

## 4. Material and Methods

All chemicals were purchased from Roth (Karlsruhe, Germany) and Sigma-Aldrich (Vienna, Austria) unless stated differently.

### 4.1. Cultivation of Micrasterias denticulata and Lemna sp.

The cells of *Micrasterias denticulata* Bréb. were cultivated in Erlenmeyer flasks, containing 30 mL of Desmidiacean medium [62] and were exposed to a light/dark cycle of 14/10 h at +20 °C in an incubator. *Micrasterias* cells were subcultured every 3 to 4 weeks.

The aquatic freshwater plant *Lemna* sp. L. was cultivated in Erlenmeyer flasks, containing 50 mL of Hoagland’s Medium [63] under axenic conditions and a light/dark cycle of 14/10 h at +20 °C. *Lemna* plants were subcultured every 5−6 weeks by transferring two single plants into new Erlenmeyer flasks with Hoagland’s medium.

The light intensity for the cultivation of the two organisms was between 100 and 150 µmol photons·m^−2^·s^−1^.

Cell vitality assays revealed that −2 °C extracellular freezing was sublethal for *Micrasterias* (data unpublished). By considering this fact, we simulated cold and freezing stress in the laboratory for our main model system *Micrasterias.* Sublethal low temperature ranges for the higher aquatic plant *Lemna* were chosen according to a previous study [33] and by observation of the recovered plants after freezing (data unpublished).

### 4.2. Field Sampling of Ranunculus glacialis

*Ranunculus glacialis* L. plants were taken from the summit area of the “Kleiner Isidor” in the Stubaier Alps (Innsbruck, Austria, 46°58’24,71” N, 11°06’27,88” E) at an elevation of 3150 m a.s.l. Individuals were dug out with roots and surrounding soil and were safely prepared for transportation. After sampling, *R. glacialis* plants were transferred in a cooling box to the laboratory in Salzburg for TEM (transmission electron microscopy) preparation and to the laboratory in Innsbruck for gas exchange measurements. In both laboratories, plants were stored up to 48 h in a climate chamber (day/night, 14/10 h) at +20 °C respectively approx. +1 °C (day/night) in order to exclude artificial ultrastructural damage from excavation and transport. Based on previous studies [44,45,46,59], +10 °C was chosen as the control temperature for *R. glacialis*. During daylight but without direct sun exposure, plant canopy temperatures of nival plants of +10 °C are highly frequent [57]. Sublethal low temperature ranges for the cold adapted higher plant *R. glacialis* were chosen according to previous studies [50,64].

### 4.3. Simulation of Chilling and Freezing in Automatic Freezing Units and Definition of Temperature Ranges

For sample preparation and implementation of preliminary cell vitality assays at defined temperatures during chilling and freezing stress, two conventional laboratory freezers (PLTA 0986, National Lab, Mölln, Germany) were rebuilt and modified to automatic freezing units (AFUs) for controlled low temperature exposure (for detailed description see [33]). The original lid of each AFU was replaced by an insulated detachable transparent Plexiglas^®^ pane. The top unit of one AFU (made from thermally insulated material) had two holes through, with thermally insulted gloves. The insulated gloves enabled working inside the AFU. Temperature inside the AFU was regulated with two ventilated heating elements (SUNON, Kaohsiung, Taiwan; DBK David and Baader, Rülzheim, Germany) and was controlled via self-developed software application [33]. The AFUs allowed for the controlled chilling and freezing of the samples prior to high pressure freezing (HPF) for electron microscopy, preparation of samples, and long-term-exposures to low temperatures.

Three temperature scenarios were used in this study. Low temperatures between +4 °C and +0.5 °C were defined as “chilling stress”. Sub-zero temperatures without extracellular ice formation were termed as “freezing stress (without ice)” and sub-zero temperatures, where samples were extracellularly frozen as measured by exothermic warming, as “freezing stress”. Temperature measurements were performed as in a previous study [33].

### 4.4. Freezing and Thawing Experiment in R. glacialis

In order to investigate physiological and structural changes during and after low temperature and freezing stress, *R. glacialis* plants were kept at +10 °C for two hours and were then transferred into the automatic freezing unit (AFU). The petioles of *R. glacialis* leaves were stored in Eppendorf tubes, filled with tap water and ice nucleating active bacteria (INA, *Pseudomonas syringae*) to promote ice nucleation within the experimental low temperature range (−2.5 °C to −3 °C). *R. glacialis* was cooled down to −5 °C in the AFU at a rate of −3 °C∙h^−1^. According to previous studies [50,64], it is known that −5 °C is sublethal for *R. glacialis*. Ice nucleation was ensured by the detection of the exothermal temperature increase in the leaves (see also [33]). Freezing was induced between −2.5 °C and −3 °C by transferring small amounts of ice crystals to the petioles of *R. glacialis*. After temperature stabilization at −5 °C, samples remained frozen for 1 h at −5 °C. Afterwards, *R. glacialis* was immediately transferred to +10 °C. Gas exchange measurements and ultrastructural analysis (TEM) were performed at +10 °C for controls, at +10 °C (15 min after −5 °C, extracellularly frozen) and at +10 °C (24 h recovery after −5 °C, extracellularly frozen). In addition, extracellularly frozen *R. glacialis* leaves at −5 °C were cryofixed for TEM analysis. Gas exchange measurements were not implementable with −5 °C extracellularly frozen *R. glacialis* leaves due to the experimental setting. The experiments were performed with one and the same plant and were replicated with 5 individual plants of *R. glacialis* (*n* = 5).

Standard gas exchange parameters such as A (carbon assimilation rate), R_d_ (dark respiration rate) and G_H20_ (diffusive conductance of water vapor) were determined by a GFS3000 Gas Exchange Measurement System (Walz, Effeltrich, Germany) inside of a controllable cooling chamber (see also [64]). During the experiment, irradiation (PPFD) was 500 µmol photons m^−2^∙s^−1^ to keep the stomata open and thus to allow the determination of R_d_ with sufficient accuracy. To minimize the effects of different stomatal opening on R_d_, the quotient R_d_/G_H2O_, was calculated. For statistical analysis, a repeated measures ANOVA with Bonferroni-correcture was applied. Statistical measurements were carried out with SPSS-software (IBM SPSS V.26.0, SPSS Inc., Armonk, NY, United States) and a significance level of α = 0.05.

### 4.5. Polarographic Oxygen Measurement

The measurements of photosynthetic oxygen evolution of +4 °C exposed *Micrasterias* cells and *Lemna* plants as well as controls at +20 °C were performed by polarographic oxygen determination (Hansatech, King’s Lynn, England). For each experiment, three biological replicates were used (*n* = 3). 6 light- (approximately 200 µmol photons·m^−2^s^−1^) and 6 dark cycles were measured in order to obtain insight into their respiratory and photosynthetic efficiency during low temperature exposure (+4 °C chilling stress) compared to +20 °C standard conditions. *Micrasterias* cells (approximately 1000 cells per mL) and single *Lemna* plants were measured during chilling stress at +4 °C after 1 h, 24 h and 3 weeks. O_2_ measurements of *Micrasterias* were performed according to earlier experiments [65,66] and were adapted for *Lemna*. For statistical analysis of variance, a one-way ANOVA was applied with additional Duncan’s post-hoc test (photosynthesis in *Micrasterias*) or Games–Howell’s post-hoc test (respiration in *Micrasterias*; photosynthesis and respiration in *Lemna*) with SPSS-software and a significance level of α = 0.05.

### 4.6. Preparation for TEM and FIB-SEM

Control temperature and low temperature samples of *Micrasterias, Lemna* and *R. glacialis* were transferred into specimen holder for high-pressure freeze fixation. *Micrasterias* cells were packed in cotton fibres (for detailed method see [67]), for transfer into the specimen holder. Leaf samples of *Lemna* and *R. glacialis* were cut out with a punching tool (item Nr. 706892, Leica Microsystems, Vienna, Austria) in the exact diameter of the specimen holder for HPF. HPF was implemented with a Leica EMPACT HPF device (Leica Microsystems, Vienna, Austria) and a cooling rate of at least 12,000 °C/s at 2040 bar and subsequently the samples were cryo-substituted and embedded as described earlier [22,25,68].

The preparation of *Micrasterias* for FIB-SEM tomography was achieved in the same way, until the last embedding step [41]. For this, the cells were smoothened on micro-scale microscope slices (neoLab Migge GmbH, Heidelberg, Germany) until single cells were coated by only a thin layer of epoxy resin. Microscope slides were cut into smaller pieces, mounted on stubs and coated with carbon to enable lateral milling via FIB (Ga-ion beam).

Sectioning for TEM-imaging was performed with a Leica UC7 Ultramicrotome (Leica Microsystems, Vienna, Austria). Ultrathin sections were collected on Formvar coated copper grids.

### 4.7. 2-D TEM and 3-D FIB-SEM Tomography

2-D TEM was carried out in a LEO 912 AB Omega TEM (Zeiss, Oberkochen, Germany) at 80 kV. The images were filtered at zero energy loss and recorded with a TRS 2k Slow-Scan CCD camera (Tröndle Restlicht Verstärker Systeme, Moorenweis, Germany).

The “slice and view” technique was carried out at a Zeiss Auriga 40 crossbeam workstation (Carl Zeiss Microscopy, Oberkochen, Germany) to obtain tomographic datasets. FIB milling was performed with 2−5 nA milling current of the Ga-emitter. The slice thickness was chosen between 10−16 nm. SEM micrographs of the block faces were taken with an aperture of 60 µm in high-current mode at +0.5 kV of the in-lens EsB detector. The alignment (semi-automatically) of the FIB/SEM image series and the segmentation (manually) was done with Amira™ (Thermo Fisher Scientific).

### 4.8. Statistical Analysis of Mitochondrial Aggregation

In order to determine the frequency of mitochondrial aggregation, mitochondrial fusions and contacts with other mitochondria were analyzed and counted in the alga *Micrasterias*. Due to the large cell size of the alga, for the counting only one fourth of each single cell was analyzed via TEM implementation. In total, ten *Micrasterias* control cells at +20 °C were compared to ten cells after −2 °C ice induction (*n* = 10). Statistical data analysis by t-Test of independent samples was performed with SPSS-software. For the statistical analysis, a significance level of α = 0.05 was used.

## 5. Conclusions

Our 2-D and 3-D electron microscopic investigations in correlation with physiological assays provided new insights into the adaptation strategies of contrasting plants to cope with chilling and freezing stress. To our knowledge, these are the first ultrastructural investigations and 3-D reconstructions of pre-frozen plant cells [24], depicted and visualized by electron microscopic methods after HPF [33]. Working with three different plant model systems showed good concordance of the results obtained, although the evolutionary level and the adaptation to low temperatures varied. Only slight variations between structural and physiological cold stress responses were observed. These differences may be due to species specific genetic temperature acclimation [69,70] but also to considerable differences in the cell size and correspondingly, in the number of organelles. Whereas *Micrasterias* cells have a diameter of 200 µm, parenchyma cells of *Lemna* measure approximately 30 µm and palisade parenchyma cells of *R. glacialis* approximately 27 µm in diameter. This means that in the large *Micrasterias* cell, with hundreds of mitochondria, fusion of these organelles is much more favorable for maintaining respiration during cold stress than in *Lemna* and *R. glacialis,* which contain only few mitochondria per cell.

Nevertheless, our results indicate that the formation of organelle networks with decreasing temperature contributes to cold stress management of plants at least during the time period when energy balance of the cells is still positive. Future studies, focusing on electrophysiology of mitochondrial membranes and ultrastructural investigations of the potential surface distribution of mitochondria during cold stress might contribute to an even broader understanding of mitochondrial adaptation mechanisms during cold stress. Moreover, the freezing and thawing experiments in *R. glacialis* show that in addition to organelle interactions, the occurrence of autophagy appears to be essential for surviving freezing stress, probably by eliminating damaged cytoplasmic constituents and thus providing a source for the re-establishment or maintenance of the cellular metabolism. In summary, both mitochondrial networking and autophagic processes appear to be important cellular mechanisms for plants to maintain the energy to withstand physiological stress during chilling and freezing events.

## Figures and Tables

**Table 1:** Overview of most prominent ultrastructural alterations of organelles in *Micrasterias, Lemna* and *R. glacialis* during cold stress in comparison to untreated controls.

Object	Mitochondria	Degradation Processes	Dictyosomes	ER and Peroxisomes	Chloroplast
***Micrasterias denticulata,*** **+20 °C control**	spherical shaped, solitary distributed	not observed	defined number of 11 cisternae, regular vesicle production	regular, unbloated ER	regular structure
***Micrasterias denticulata,*** **+4 °C chilling stress**	elongated, occasional aggregation and fusion, protrusion into mucilage vesicles	not observed	defined number of 11 cisternae, regular vesicle production	slightly bloated ER	regular structure
***Micrasterias denticulata,*** **−2 °C freezing stress (without ice)**	elongated, fusion and aggregation to local mitochondrial networks	not observed	defined number of 11 cisternae, regular vesicle production	slightly bloated ER	bloated thylakoids
***Micrasterias denticulata,*** **−2 °C freezing stress**	elongated, aggregation and fusion to local mitochondrial networks, protrusion into mucilage vesicles	not observed	degradation of cisternae, reduced production of vesicles	bloated ER, protrusion of peroxisomes into mucilagevesicles	bloated thylakoids
***Lemna* sp.,** **+20 °C control**	spherical shaped, solitary distributed	not observed	no alterations of cisternae, regular vesicle production	regular, unbloated ER	regular structure
***Lemna* sp.,** **+4 °C chilling stress**	elongated, occasional aggregation and fusion	not observed	no alterations of cisternae, regular vesicle production	slightly bloated ER	regular structure, slightly enlarged starch grains
***Lemna sp.,*** **−2 °C freezing stress**	elongated, occasional aggregation and fusion	numerous autophagic structures	strong degradation of cisternae	bloated ER	dissolved chloroplast membrane, large starch grains and single thylakoids remain in cytoplasm
***Ranunculus glacialis,*** **+10 °C control**	spherical shaped, solitary distributed	not observed	no alterations of cisternae, regular vesicle production	regular, unbloated ER	regular structure
***Ranunculus glacialis,*** **−5°C freezing stress**	elongated, degradation of cisternae, aggregation and fusion	numerous autophagic structures	strong degradation of cisternae	bloated and enlarged ER	degraded outer membrane, degraded thylakoid structure
***Ranunculus glacialis,*** **+10 °C, 15 min after −5 °C freezing stress**	elongated, slight degradation of cisternae, aggregation and fusion	multi vesicular bodies	not observed	bloated and enlarged ER	degraded outer membrane, degraded thylakoid structure
***Ranunculus glacialis,*** **+10 °C, 24 h after −5 °C freezing stress**	elongated	not observed	no alterations of cisternae, regular vesicle production	slightly bloated ER	regular structure

## Abbreviations

TEM	Transmission Electron Microscopy
FIB-SEM	Focussed Ion Beam-Scanning Electron Microscopy
HPF	High Pressure Freezing
AFU	Automatic Freezing Unit
PPFD	Photosynthetic Photon Flux Density
ER	Endoplasmic Reticulum
PCD	Programmed Cell Death
